# Regulating thermosalient behaviour in three polymorphs

**DOI:** 10.1107/S2052252517005577

**Published:** 2017-04-25

**Authors:** Maximilian J. Werny, Jagadese J. Vittal

**Affiliations:** a Department of Chemistry, National University of Singapore, 3 Science Drive 3, 117543, Singapore

**Keywords:** polymorphism, intermolecular interactions, thermosalient effect, dynamic crystals

## Abstract

Three polymorphs of a di­chloro-*N*-salicylideneaniline derivative show visually impressive jumping and sudden blasting behaviours on heating due to phase transitions.

Dynamic molecular crystals can be defined as crystalline materials that respond to external stimuli with mechanical responses on a macro-, micro- or nanoscopic scale (Naumov *et al.*, 2015 ▸). The motility of dynamic crystals is usually triggered by a phase transition or chemical reaction without gaseous products, subsequently creating local stresses in the densely packed crystal lattice of the respective material (Commins *et al.*, 2016 ▸). The amplification of such strain *via* cooperative processes can lead to reversible or irreversible phase deformation, instantaneous propulsion and even disintegration. Crystalline materials, which are classically defined as rigid and brittle entities, thus display a reversible mechanical response in the form of shape change (curling, bending, twisting) or locomotion (jumping, flipping, rotation, *etc*.) (Nath *et al.*, 2014 ▸).

A class of dynamic single crystals, known as thermosalient crystals, can propel themselves over distances hundreds of times larger than their own size when they are exposed to heat. The earliest known thermosalient crystal, (phenyl­azo­phenyl)­palladium hexa­fluoro­acetyl­acetone, was reported more than 30 years ago (Etter & Siedle, 1983 ▸). Only in recent years, however, have researchers begun to fully investigate thermosalient phase transitions on a structural, kinematic and mechanistic basis. The observed conversion of heat energy into mechanical motion by a densely packed crystal lattice represents a phenomenon that could be integrated into a host of new applications, *e.g*. actuators, sensors and pressure-sensitive applications (Commins *et al.*, 2016 ▸).

In general, a limited yet distinct anisotropic expansion of the crystal unit cell is accompanied by the nucleation and propagation of a new phase within the bulk crystal (phase transition) (Nath *et al.*, 2014 ▸). The resulting build-up of strain at the phase interface, if larger than the cohesive interactions present within the crystal lattice, instigates a mechanical force (self-actuation).

Phenomena such as molecular dimerization, intermolecular charge transfer and intermolecular proton transfer depend on the way the molecules are assembled in a crystal. Similarly, the dynamic properties of crystals, which occur in response to external stimuli, are also controlled by the intermolecular interactions. The *π*–*π* interactions, hydrogen bonds, halogen bonds and coordination bonds contribute decisively to the cooperative behaviour of the dynamic switching process. In the case of thermoresponsive materials, the dissipation of thermal energy is influenced by the crystal packing (Naumov *et al.*, 2015 ▸).

The study of a di­chloro derivative of *N*-salicylideneaniline (see Fig. 1 ▸) by Mittapalli *et al.*, reported in this issue of **IUCrJ**, revealed four polymorphs, three of which exhibit mechanical responses such as jumping (forms I and III) and exploding (form II) during phase transition at high temperature (Mittapalli *et al.*, 2017 ▸). This is a rare example of a combination of mechano­physical responses in polymorphs of the same compound (Steiner *et al.*, 1993 ▸; Crawford *et al.*, 2007 ▸; Sahoo *et al.*, 2013 ▸). The crystal structures showed that the *N*-salicylidene­aniline molecules interact *via* amide N–H⋯O dimer-synthon and C–Cl⋯O halogen-bond motifs in all polymorphic modifications. The type of thermomechanical response was found to correlate with the packing structure. While forms I and III display layered sheet morphology that leads to thermal stress being transmitted in a single direction, the thermodynamically more stable form II is characterized by a corrugated or wave-like sheet structure, which in turn causes a more isotropic yet sudden thermal release.

Mittapalli *et al.* also concluded that the presence of the weak C–Cl⋯O halogen bond interactions is crucial for structural dynamics. Isostructural Br and I derivatives did not display a mechanical response upon heating, as the halogen bonds are considerably stronger here. Weak halogen bonding, as in the case of C–Cl⋯O, permits conformational flexibility while maintaining the network connectivity. Only slight changes in the halogen–halogen bond distances were observed during phase transition. The above examples prove that controlled modification can help to understand the relationship between structure and properties. It is evident that the weak halogen bond serves as yet another structure-directing motif, concurrently controlling the degree of stress accumulation and release during thermal expansion. Thus, weaker interactions must also be considered in the design of novel thermosalient materials. Indeed, Desiraju’s laboratory have demonstrated that mechanical bending in organic crystals can be engineered *via* halogen–halogen interactions (Reddy, Padmanabhan *et al.*, 2006 ▸; Reddy, Kirchner *et al.*, 2006 ▸; Mukherjee *et al.*, 2014 ▸). The work described by Mittapali *et al.* highlights another facet of the weak halogen-bond interactions.

Dynamic processes induced by external stimuli include spin transition, charge transfer, proton transfer, and change in the molecular conformation and structure (Sato, 2016 ▸; Mishra *et al.*, 2015 ▸; Williams, 2015 ▸). The same crystalline material can respond in two different ways to two different external stimuli. For example, the sudden movements of crystals of a Co(III) complex have been accompanied by the isomerization of nitro groups to nitrito groups (Naumov *et al.*, 2013 ▸) under UV radiation. Interestingly, the single crystals of this coordination complex can also be bent under UV light (Boldyreva, 2001 ▸). It is worthwhile noting that the single crystals of salicylidene­aniline derivatives can be bent by UV light, and these compounds can also exhibit photochromism *via* keto–enol tautomerism (Ghosh *et al.*, 2015 ▸; Koshima *et al.*, 2011 ▸). It would be interesting to see if the thermosalient materials described here also show these properties, thus giving rise to multi-functional mechanical effects.

## Figures and Tables

**Figure 1:**
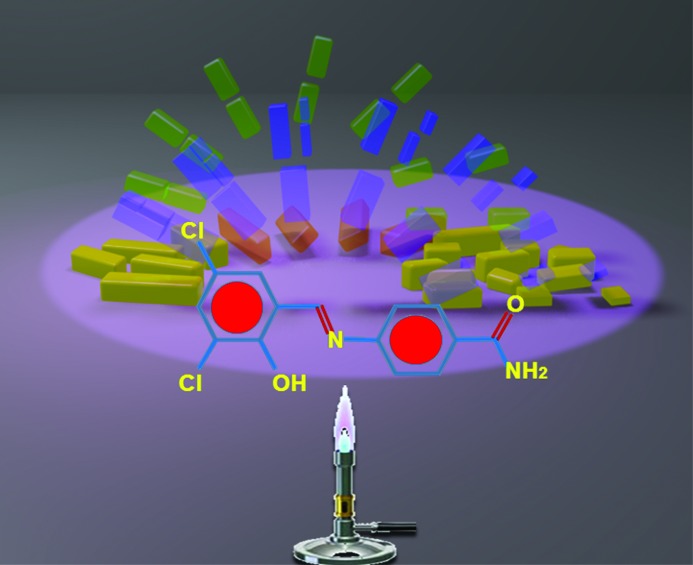
Thermosalient effects demonstrated by the single crystals of polymorphs of di­chloro-*N*-salicylideneaniline.
